# POTENTIALLY INAPPROPRIATE MEDICATION USE INCREASES RISK OF INCIDENT DISABILITY IN HEALTHY OLDER ADULTS

**DOI:** 10.1093/geroni/igac059.2728

**Published:** 2022-12-20

**Authors:** Jessica Lockery, Taya Collyer, Robyn Woods, Suzanne Orchard, Anne Murray, Michael Ernst

**Affiliations:** RMIT University, Melbourne, Victoria, Australia; Monash University, Melbourne, Victoria, Australia; Monash University, Melbourne, Victoria, Australia; Monash University, Melbourne, Victoria, Australia; Hennepin Healthcare, Minneapolis, Minnesota, United States; The University of Iowa, Iowa City, Iowa, United States

## Abstract

**Background:**

Efforts to minimize medication risks among older adults include avoidance of potentially inappropriate medications (PIMs). However, most PIMs research has focussed on aged or inpatient care, creating an evidence gap for community-dwelling older adults. To address this, we investigated the impact of PIMs use in the ASPREE clinical trial.

**Methods:**

ASPREE enrolled 19,114 community-dwelling participants aged 70+ years (65+ if US minorities) without a history of major cardiovascular disease, cognitive impairment, and significant physical disability. PIMs was defined according to a modified 2019 AGS Beers Criteria. Cox proportional-hazards regression models were used to estimate the association between baseline PIMs exposure, and disability-free survival, death, disability, and hospitalization, with adjustment for comorbidities including frailty.

**Results:**

At baseline, 7396 (39% of total) participants were prescribed at least one PIM. Compared with those unexposed, participants on a PIM at baseline were at an increased risk of persistent physical disability (Adjusted HR 1.47, 95%CI 1.21, 1.80) and hospitalization (Adjusted HR 1.26, 95%CI 1.20, 1.32), but had similar rates of disability free survival and death. These effects did not vary by polypharmacy status. PIMs exposure was associated with higher risk of disability followed by hospitalization (Adjusted HR 1.92, 95%CI 1.25, 2.96) as well as vice versa (Adjusted HR 1.54, 95%CI 1.15, 2.05). Conclusions: PIMs exposure is associated with increased risk of incident disability and hospitalization. Increased risk of disability prior to hospitalization suggests that PIMs use may start the disability cascade, emphasizing the importance of caution when prescribing PIMs for community-dwelling older adults.

